# The Impact of Low-Level Benzalkonium Chloride Exposure on *Staphylococcus* spp. Strains and Control by Photoinactivation

**DOI:** 10.3390/antibiotics12081244

**Published:** 2023-07-28

**Authors:** Erika C. R. Bonsaglia, Gustavo H. Calvo, Daniel O. Sordelli, Nathalia C. C. Silva, Vera L. M. Rall, Adriana Casas, Fernanda Buzzola

**Affiliations:** 1 Instituto de Investigaciones en Microbiología y Parasitología Médica (IMPaM), Facultad de Medicina, Universidad de Buenos Aires, Consejo Nacional de Investigaciones Científicas y Técnicas, Buenos Aires 1121, Argentina; ebonsaglia@fmed.uba.ar (E.C.R.B.); gustavohcalvo@gmail.com (G.H.C.); sordelli@fmed.uba.ar (D.O.S.); 2 Department of Food Science, Faculty of Food Engineering (FEA), University of Campinas (UNICAMP), Campinas 13083-862, Brazil; ncirone@unicamp.br; 3 Department of Chemical and Biological Sciences, Institute of Biosciences, Sao Paulo State University, Botucatu 18618-691, Brazil; vera.rall@unesp.br; 4 Centro de Investigaciones sobre Porfirinas y Porfirias (CIPYP), Hospital de Clínicas José de San Martín, Universidad de Buenos Aires, Consejo Nacional de Investigaciones Científicas y Técnicas, Buenos Aires 1121, Argentina; adriana@qb.fcen.uba.ar

**Keywords:** *Staphylococcus* sp., antimicrobial resistance, photodynamic inactivation

## Abstract

Exposure of bacteria to low concentrations of biocides can facilitate horizontal gene transfer, which may lead to bacterial adaptive responses and resistance to antimicrobial agents. The emergence of antibacterial resistance not only poses a significant concern to the dairy industry but also adds to the complexity and cost of mastitis treatment. This study was aimed to evaluate how selective stress induced by benzalkonium chloride (BC) promotes antibiotic non-susceptibility in *Staphylococcus* spp. In addition, we investigated the efficacy of photodynamic inactivation (PDI) in both resistant and susceptible strains. The study determined the minimum inhibitory concentration (MIC) of BC using the broth microdilution method for different *Staphylococcus* strains. The experiments involved pairing strains carrying the *qac*A/*qac*C resistance genes with susceptible strains and exposing them to subinhibitory concentrations of BC for 72 h. The recovered isolates were tested for MIC BC and subjected to disc diffusion tests to assess changes in susceptibility patterns. The results demonstrated that subinhibitory concentrations of BC could select strains with reduced susceptibility and antibiotic resistance, particularly in the presence of *S. pasteuri*. The results of PDI mediated by toluidine blue (100 µM) followed by 60 min irradiation (total light dose of 2.5 J/cm^2^) were highly effective, showing complete inactivation for some bacterial strains and a reduction of up to 5 logs in others.

## 1. Introduction

Intramammary infection in cows caused by *Staphylococcus* spp. is the most concerning disease that affects the dairy chain, causing vast economic losses worldwide. Bovine mastitis is conditioned by diverse factors and, for this reason, requires specific treatment according to the infecting agent, thus making disease control even more difficult, generating high costs for the producer [1]. *S. aureus* and *Staphylococcus non-aureus* showing resistance to antibiotics and biocides have been isolated in the dairy chain. It is known that the inappropriate use of antimicrobial agents in the dairy industry has contributed to the emergence of multi-resistant pathogens [2].

Antimicrobial agents and biocides have a wide range of applications worldwide, and are an important tool for management of unwanted bacterial growth. However, antibiotic and biocide effectiveness is decreasing due to the emergence of resistance through different mechanisms across different lineages and species. Indeed *Staphylococcus* spp. has a remarkable ability to acquire resistance to antimicrobial agents, either through mutation of chromosomal bacterial genes or through the incorporation of resistance genes through transfer from other microorganisms [3]. It has been proposed that there is a link between antibiotics and resistance to disinfectants such as quaternary ammonium compounds (QACs). In fact, QACs are frequently used in the food industry, including dairy environments, for disinfection of the environment and equipment due to their broad antimicrobial spectrum against bacteria, fungi, and viruses. Many QAC resistance genes (*qacA*, *qacB*, *smr*, *qacG*, *qacH*, *qacJ*) have already been identified in bacteria isolated from different sources. Due to their location on mobile genetic elements, the interaction between different species of *Staphylococcus* is facilitated [2]. QAC efflux pump determinants are usually found on multidrug resistance plasmids in *Staphylococcus spp*. strains. In this regard, previous studies have found the *qacA/B* genes located on the same plasmid that confers resistance to β-lactams in clinically- and in food-derived strains with the potential for uptake by plasmid-free *S. aureus*, indicating its ability to transfer under selective stress [4,5].

In the current scenario, the emergence of multi-resistant strains becomes a challenge for the pharmaceutical industry. The recurrent problem of lack of options for controlling these pathogens makes essential to seek new therapeutic alternatives and to review practices that may influence the emergence of antibiotic-resistant bacteria. Photodynamic inactivation (PDI), also known as antimicrobial photodynamic therapy, is an emerging therapy that involves the use of a photosensitizer agent with specific wavelength light to generate reactive oxygen species with the goal of destroying microbial agents. This procedure lacks genotoxic and mutagenic effects, preventing the development of bacterial resistance, and had exhibited excellent results in pathogens isolated from bovine mastitis [6].

The present study demonstrates that exposure to benzalkonium chloride (BC) subinhibitory concentrations select *S. aureus* strains with reduced susceptibility, and also confers an increase in antibiotic resistance. In addition, we evaluate and underline the effects of PDI on mutant strains that presented multi-resistance to antibiotics and BC.

## 2. Results

### 2.1. Exposure to Subinhibitory Concentration of Benzalkonium Chloride

**Figure 1:**
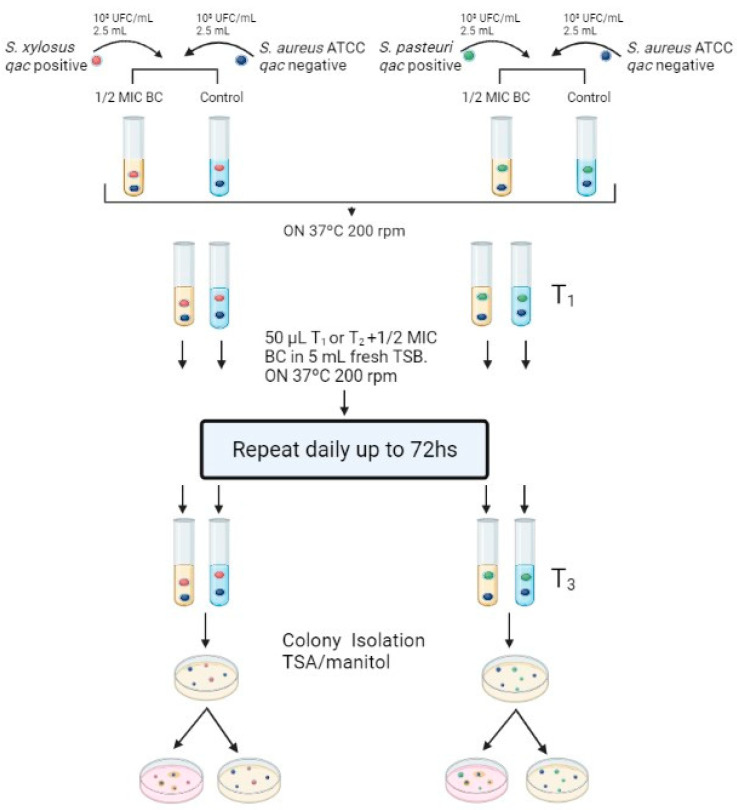
Schematic representation of benzalkonium chloride exposure assay. *S. xylosus* carries *qacA/B* and *mecA* genes, and *S. pasteuri* carries *qacC* gene. *S. aureus* ATCC6538 and ATCC29213 strains are susceptible to BC and antibiotics. T1: 24 h post co-culture. T2: 24 h post subculture of T1. T3: 24 h post subculture of T2. Created in BioRender.com.

**Figure 2:**
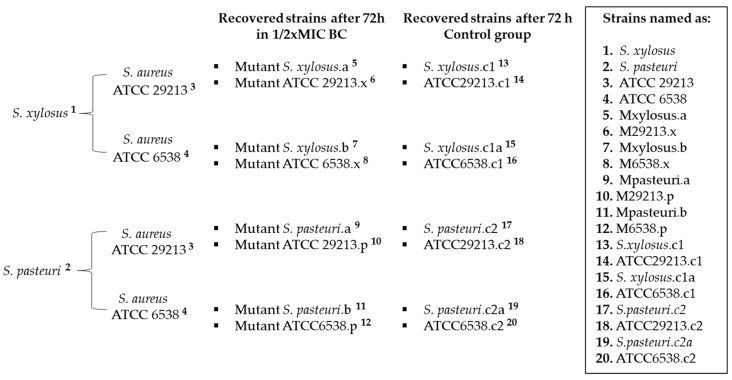
Nomenclature used for the strains and control group recovered after 72 h of exposure to subinhibitory concentration of benzalkonium chloride.

Resistant strains of *S. xylosus* and *S. pasteuri* were, respectively, paired with the susceptible strains of *S. aureus* ATCC29213 and ATCC6538 (Figure 1). All tested strains were recovered after 72 h of passage in a 1/2 × MIC of BC and were renamed according to Figure 2. Control group strains were also recovered after 72 h.

### 2.2. Agar Diffusion and MIC of Benzalkonium Chloride

After BC sub-MIC assays, all strains were subjected to antimicrobial agar diffusion testing using cefoxitin, cefazolin, clindamycin, and penicillin discs. Oxacillin MIC values were determined by E-test. Table 1 shows the antimicrobial susceptibility obtained before and after exposure to sub-MIC BC. Phenotypic alteration occurred in only one strain (Table 1). Regarding the *S. aureus* strains that were grown together with *S. xylosus*, only the strain named 8 (M6538.x) showed a two-fold increase (7.8 µg/mL) in MIC of BC value compared to that of its parental ATCC6538 strain (3.9 µg/mL). Moreover, when the *S. aureus* ATCC6538 strain was confronted with *S. pasteuri*, the recovered mutant exhibited a four-fold increase in resistance (15.7 µg/mL) compared to the parental MIC of BC value (3.9 µg/mL). In addition, mutant number 10 of the *S. aureus* ATCC29213 strain (named M29213.p) showed a two-fold increase (15.7 µg/mL) of the BC MIC compared to value of its parental strain (7.8 µg/mL) (Table 1). None of the recovered strains of the control group showed alterations in MIC or antibiotic susceptibility and, for this reason, they were not included in Table 1.

### 2.3. Photodynamic Inactivation (PDI) of Planktonic Cultures

**Figure 3:**
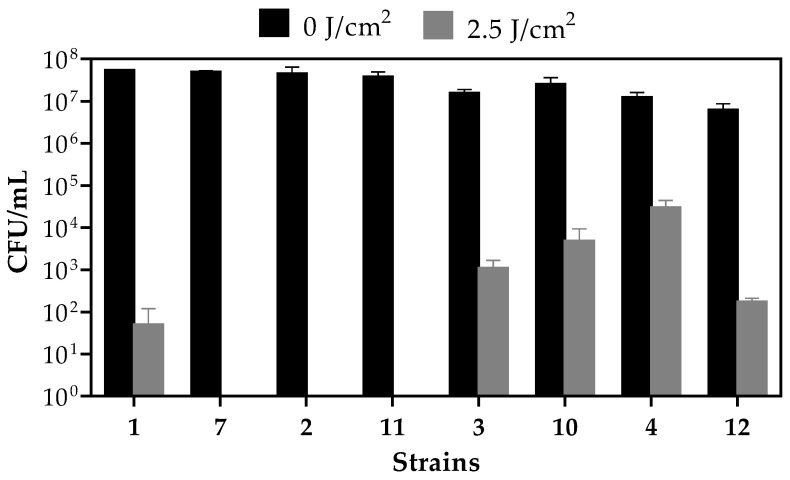
Total viable bacteria (CFU/mL) exposed to a light dose of 0 or 2.5 J/cm^2^. Strains were named as 1: *S. xylosus*; 7: *Mxylosus.b*; 2: *S. pasteuri*; 11: *Mpasteuri.b*; 3: *S. aureus* ATCC29213; 10: M29213.p; 4: *S. aureus* ATCC6538; 12: M6538.p. Control assays irradiated without TB and non-treated bacteria showed values of CFU/mL ranging from 1 × 10^7^ to 5 × 10^7^ CFU/mL. Thus, no toxic per se effect of TB was observed. Each bar represents the mean ± SD values of 2 independent experiments performed in triplicate. Statistical significance of TB-PDI (grey bars) vs. control TB non-irradiated (black bars): *p* = 0.0026, two-way ANOVA and D’Agostino–Pearson normality test *p* > 0.05.

All bacterial strains exposed to PDI mediated by TB (TB-PDI) showed a very high reduction in their populations. There were differences between the effect of the treatment among strains, but even in the strain that showed the least reduction, the number of bacteria decreased from 1.25 × 10^7^ to 3 × 10^4^ CFU/mL total bacteria, which represents a 99.76% mortality in the bacterial population (Figure 3, strain number 4). The *S. xylosus* and *S. pasteuri* strains named as 1, 7, 2 and 11 (Figure 2) were more susceptible to the TB-PDI treatment. Moreover, strains 7, 2 and 11 showed virtually total bacterial death after TB-PDI. *S. aureus* strains named 3, 10, 4 and 12 (Figure 2) appeared to be less susceptible to the treatment, although the bacterial mortality observed was around 99%. The total CFU/mL of the strains before and after treatment, and the percentage of bacterial death, are shown in Table 2.

It is worth mentioning that the three strains (named 11, 10 and 12) with high MIC of BC values were shown to be susceptible to TB-PDI. Therefore, this therapy may be useful to combat strains bearing the *qac* genes. None of the strains studied showed intrinsic photosensitivity in the absence of the photosensitizer TB.

## 3. Discussion

The growing restrictions on antimicrobial use and the expansion of multi-resistant bacteria highlight the necessity to explore alternative or complementary methods to control mastitis in dairy herds [7,8]. One of the major challenges is that *Staphylococcus* spp., besides possessing a wide array of virulence factors that hinder its elimination, also exhibits diverse responses to the presence of antimicrobial agents. Acquired resistance is one of these mechanisms, which can arise through mutations in normal genes due to external and internal factors, or by acquiring genetic information from other microorganisms, enabling the bacteria to survive in harsh environments [9]. Environmentally relevant concentrations of biocides are likely below those required to inhibit microbial growth. In our present study, we observed that susceptible strains to BC, when exposed to subinhibitory concentrations of this agent in the presence of resistant strains, exhibited changes in susceptibility not only to BC but also to several antibiotics. These findings support the evidence that prolonged use of BC may facilitate the emergence of antibiotic-resistant strains and compromise the efficacy of other antimicrobial agents, thereby diminishing the ability to control infections [10]. Quaternary ammonium-based sanitizers are commonly used to clean dairy equipment and environments. However, as our study reveals, the prolonged and/or improper use of these sanitizers can foster the development of more resilient bacterial clones, particularly in *S. aureus*. Hence, it is crucial to implement appropriate cleaning and disinfection strategies and to regularly assess the efficacy of these products to minimize the risk of selecting and disseminating resistant strains. A previous study by Weber et al. [11] has shown that the improper use or dilution of sanitizers can exert selective pressure on microorganisms, thus effectively contributing to the rise in bacterial resistance. Furthermore, the presence of these resistance genes in mobile elements, such as plasmids, enables rapid and facilitated transfer between different *Staphylococcus* species. In our study, we detected changes in susceptibility patterns of susceptible strains exposed to BC within a period as short as 72 h. Interestingly, the control group strains did not exhibit any changes, indicating that these events may occur more readily under stress conditions. However, several factors can influence the success of this transfer. Our results revealed that among the strains confronted with *S. xylosus*, only one displayed a change in susceptibility, while both *S. aureus* ATCC strains demonstrated resistance acquisition when exposed to *S. pasteuri*. This suggests that both *S. aureus* ATCC strains have good receptivity, and that *S. pasteuri* may be a more efficient donor compared to *S. xylosus*. However, to substantiate this claim, further studies involving different types of plasmids would be necessary, as the observed variability could be influenced by factors such as the specific plasmid type, the donor and recipient strains involved, as well as their interactions [12]. Based upon our findings, it can be inferred that adaptive mutations initially select strains with increased tolerance to chemical stress, subsequently leading to a selective pressure that favors the development of a cross-resistance phenotype to various other antimicrobials. The definition of tolerant strains varies among researchers. According to Gerba [13], tolerant strains are those exhibiting any increase in minimum inhibitory concentration (MIC) compared to their control. However, other authors define tolerant strains as those capable of surviving treatment without an increase in MIC, with tolerance preceding the development of resistance [14]. After conducting the strain confrontations, we observed both scenarios mentioned earlier in the mutants, with variations observed between the species involved. For instance, strain *S. aureus* ATCC29213 did not exhibit an increase in the MIC of BC when confronted with *S. xylosus*, but was able to survive for 72 h in subinhibitory concentration. However, when confronted with *S. pasteuri*, its MIC increased twice compared to its initial value. On the other hand, *S. aureus* ATCC6538 displayed an increase in MIC in both situations, with a four-fold higher increase observed in the presence of *S. pasteuri*. Notably, *S. aureus* ATCC6538 demonstrated cross-resistance potential, as it exhibited changes in resistance phenotypes to all four tested antibiotics. Therefore, we understand that tolerance may be associated with metabolic changes that enable survival in environments. For tolerant strains that do not exhibit altered antibiotic phenotypes, it is only a matter of time before they acquire cross-resistance to other agents. While all strains require an adaptation period, there are cases where the emergence of resistant strains may occur more rapidly within a population. The excessive use of antimicrobials in milk production can lead to the selection of drug-resistant bacterial strains and contribute to increased selective pressure within the bacterial population present in the environment. Consequently, there is a growing concern regarding the spread of these resistant bacteria, which can compromise milk quality and the health of animals and humans involved in the production chain. Therefore, the identification of an effective mastitis treatment that does not promote the selection of drug-resistant populations is extremely necessary. Photodynamic therapy has demonstrated remarkable efficacy in the treatment of microbial diseases in humans [15,16]. However, limited research has explored its application in bovine mastitis pathogens and drug-resistant bacteria. Silva et al. [17] evaluated the effect of photoinactivation on strains of clinical and subclinical mastitis isolated from sheep, and concluded that this treatment is promissory for bovine mastitis. Furthermore, Sellera et al. [6] demonstrated the efficacy of PDI on different antibiotic-resistant species isolated from bovine mastitis. These studies provide evidence that photoinactivation therapy holds great promise as an alternative for treating mastitis in animals, as positive outcomes have been consistently achieved irrespective of the parameters and photosensitizers employed. In our study, we have shown that TB-PDI can effectively reduce bacterial populations that were resistant to BC under experimental conditions. Strains that acquired resistance to biocides were more sensitive to TB-PDI treatment than the same strains used as controls. This might be due to the accumulation of diverse negative stimuli upon the bacterial population. Nevertheless, all the strains studied showed very high sensitivity towards PDI treatment, which positions PDI as a very promising multipurpose alternative: on one hand, to kill resistant strains of bacteria that cause mastitis in herds and that function as a resistant gene reservoir (which can be passed into non-resistant strains in certain conditions); on the other hand, to kill bacteria that acquire resistance genes from the environment. In any event, the final result will be the reduction of the total number of bacteria from animals and from equipment utilized in the dairy industry.

## 4. Materials and Methods

### 4.1. Bacterial Strains

Experiments were conducted using two reference strains *Staphylococcus aureus* ATCC 6538 and ATCC 29213. *S. aureus* ATCC6538 is conventionally used to evaluate the efficacy of biocidal agents [18], and *S. aureus* ATCC29213 represents a methicillin-sensitive strain that serves as a standard quality-control strain in laboratory testing [19]. Additionally, two wild *Staphylococcus* spp. strains isolated from dairies in previous studies [20] were used to perform the experiments: (a) *S. xylosus* which carry the *qacA/B* and *mecA* genes; and (b) *S. pasteuri* which carry the *qacC* and *lsaB* genes.

### 4.2. Determination of BC Minimum Inhibitory Concentration

The microdilution broth method was used to determine the minimum inhibitory concentration (MIC) of BC, performed according to the Clinical and Laboratory Standards Institute (CLSI) guidelines for MIC testing of *Staphylococcal* species [21]. All strains were inoculated in Mueller–Hinton broth and incubated at 37 °C for 24 h. Afterwards, cultures were standardized using the McFarland 0.5 scale (1 × 10^8^ CFU/mL) as the starting inoculate, and an aliquot of 100 µL of standardized bacteria was added. Then, the 96-well plates were coated with 100 µL serial dilutions of the sanitizer tested. The dilution ranges applied for BC spanned from 1.95 µg/mL to 250 µg/mL. Media-only aliquots were added as sterility checks and as a positive control of antibiotic-mediated killing. All test sample volumes were 200 μL/mL per well with duplicates. The plates were incubated for 24 h at 37 °C under static conditions. Following incubation, the turbidity was measured using a microplate reader (Multiskan EX, Thermo Electron Corporation, Waltham, MA, USA) at 595 nm optical density. The lowest concentrations of BC that resulted in a greater reduction in turbidity compared to the respective positive-growth controls were defined as the MIC. All MIC tests were performed in three biological replicates.

### 4.3. Benzalkonium Chloride Exposure Assays

The subinhibitory concentration (subMIC) of BC was defined based on the MIC values determined previously. The experiments were performed by confronting strains carrying the *qacA/qacC* resistance genes (*S. xylosus* and *S. pasteuri*) with sensitive strains (*S. aureus* ATCC 29213 and ATCC 6538) according to Karatzas et al. [22], with modifications. Overnight cultures were standardized to 8.5 × 10^8^ CFU/mL in 5 mL of TSB. Following this, a mixture containing 2.5 mL of each strain to be confronted (one resistant and one sensitive strain) was added to two separate tubes. One tube served as a control, while the other tube had 1/2× MIC of BC added. The tubes were incubated in agitation (200 rpm) at 37 °C for 24 h. The next day, from all tubes that showed bacterial growth, an aliquot of 50 µL was transferred to a new tube with fresh medium using the same 1/2 × MIC concentration of BC and incubated under the same conditions. These passages were performed for up to 72 h. After this period, an aliquot was plated on trypticase soy agar (TSA) and mannitol agar, incubated for 24 h, and colonies with different morphologies in TSA and mannitol were separated onto a new plate and frozen for subsequent coagulase and phenotypic testing (Figure 1). The isolates recovered after 72 h were subjected to determination of the MIC of BC to verify resistance transfer. The disk diffusion test was also performed to evaluate changes in susceptibility patterns. All bacteria were stored in TSB medium with 20% glycerol at −20 °C until use.

### 4.4. Antimicrobial Susceptibility Testing by Agar Disk Diffusion

Agar disk diffusion was performed in all strains before and after of subMIC BC exposure assays using clindamycin (2 µg), cefalotin (30 µg), penicillin (10 U) and cefazolin (30 µg) according to CLSI standards [21]. In addition, oxacillin MIC were determined using E-test strips (Liofilchem, MTS^TM^, Roseto degli Abruzzi, Italy) with a 0.5 McFarland standard inoculum on Mueller–Hinton agar plates (Britania, Buenos Aires, Argentina) according to the manufacturer’s manual.

### 4.5. Light Source

An array of three white, fluorescent lamps (Osram, Buenos Aires, Argentina) was employed as a non-coherent light source, with an emission spectrum ranging from 400 to 700 nm. Light power was measured with a Yellow Springs Kettering 65 radiometer (Yellow Springs, OH, USA). The light dose of 2.5 J/cm^2^ was obtained by applying 60 min of light exposure.

### 4.6. Photosensitizer

Toluidine blue (TB) (Sigma Chem. Co., St. Louis, MO, USA) was employed as a photosensitizer at a concentration of 100 µM for treatment of bacterial suspensions.

### 4.7. Photoinactivation of Planktonic Cultures

To evaluate the efficacy of TB-PDI, we chose the strains that showed the greatest changes in susceptibility patterns to BC and antibiotics (Table 1). From an overnight culture, a tube containing 10 mL of TSB with a bacterial concentration of O.D. 0.05 was prepared and incubated at 37 °C under constant agitation of 200 rpm until reaching an O.D. 0.2. An aliquot of 10 µL was taken for control dilutions of inoculum. Then, the bacterial suspensions were centrifuged at 10,000 rpm for 10 min at 4 °C, washed and suspended in 10 mL of sterile PBS. A volume of 1 mL of each bacterial suspension was added to a tube and incubated with TB in darkness for 30 min at room temperature. After this period, aliquots of 200 µL of each culture were plated by duplicate in a 96-well microplate. Then, the plate placed on a glass slide was irradiated for 60 min from below at 16 cm from the light source, reaching a maximum light dose of 2.5 J/cm^2^. The viable bacteria cell number was determined by quantitative plating on TSA, the experimental condition consisted of bacterial suspension treated with TB and exposed to light. The following conditions were used as controls: (1) bacterial suspension treated with TB without receiving light; (2) non-TB treated bacterial suspension exposed to light; and (3) untreated bacterial suspension.

### 4.8. Statistical Analysis

For TB-PDI statistical analysis, a D’Agostino–Pearson normality test and a two-way ANOVA were performed using the GraphPad Prism 8 software, and *p* < 0.05 values were considered statistically significant.

## 5. Conclusions

Based on our study, the use of subinhibitory concentrations of BC in dairy environments can lead to an increased dissemination of resistance, promoting cross-resistance transfer and further exacerbating the issue of antibiotic resistance. However, we observed that the photodynamic therapy (TB-PDI) can be a promising alternative to control the spread of strains with decreased sensitivity to biocides and antibiotics, as well as a treatment option for mastitis, irrespective of the phenotype and species involved.

## Figures and Tables

**Table 1:** Susceptibility to antimicrobial agents and benzalkonium chloride before (grey) and after subinhibitory concentration exposition.

Strains	MIC BC µg/mL	MIC Oxacilin µg/mL	Clindamicin	Cefalotin	Cefazolin	Penicillin
1. *S. xylosus*	7.8	6	R	S	S	S
5. Mxylosus.a	7.8	6	R	S	S	S
7. Mxylosus.b	7.8	6	R	S	S	S
2. *S. pasteuri*	7.8	6	R	S	S	S
9. Mpasteuri.a	15.7	6	R	S	S	S
11. Mpasteuri.b	15.7	6	R	S	S	S
3. ATCC 29213	7.8	0.125	S	S	S	S
6. M29213.x	7.8	0.125	S	S	S	S
10. M29213.p	15.7	0.125	S	S	S	S
4. ATCC 6538	3.9	0.094	S	S	S	S
8. M6538.x	7.8	0.094	S	S	S	S
12. M6538.p	15.7	0.094	R	R	R	R

BC: Benzalkonium chloride; R: resistant; S: susceptible.

**Table 2:** Effect of TB-PDI on bacterial survival of the different strains studied.

Strain	CFU/mL		% Death
	No Light	Light	
1	5.5 × 10^7^	5 × 10^1^	99.99
7	5 × 10^7^	0	100
2	4.55 × 10^7^	0	100
11	3.8 × 10^7^	0	100
3	1.55 × 10^7^	1.1 × 10^3^	99.99
10	2.5 × 10^7^	4.8 × 10^3^	99.98
4	1.25 × 10^7^	3 × 10^4^	99.76
12	6.25 × 10^6^	1.75 × 10^2^	99.99

## Data Availability

Data will be made available upon request.
